# Protective effect of nicotinamide and l-arginine against monocrotaline-induced pulmonary hypertension in rats: gender dependence

**DOI:** 10.1007/s43440-020-00125-y

**Published:** 2020-07-06

**Authors:** Katarzyna Sztormowska-Achranowicz, Zbigniew Jankowski, Ivan Kocić

**Affiliations:** 1 Department of Pharmacology, Medical University of Gdansk, Gdańsk, Poland; 2 Department of Forensic Medicine, Medical University of Gdansk, Gdańsk, Poland

**Keywords:** Gender, Monocrotaline, Nicotinamide, l-Arginine, Pulmonary arterial hypertension, Heart contractility

## Abstract

**Background:**

The purpose of this paper was to examine the effects of nicotinamide (ND) and l-arginine (l-ARG) on pulmonary vascular and heart changes induced by pulmonary hypertension in rats in a gender-dependent way.

**Methods:**

Experiments were performed on male (M) and female (F) rats. PAH was induced via monocrotaline injection (sc, 60/kg B.W.) on day one of the 23-day observational period. After that, the animals were sacrificed, hearts removed and weighed and the papillary muscles isolated to measure force of contraction (*F*_c_). Morphological changes of pulmonary vessels were also examined.

**Results:**

Mixed diet supplementation with l-ARG + ND prevented highly significant right ventricle enlargement induced by PAH in both, male and female rats. Weight ratios between the right ventricle (RV) on one side and the left ventricle with septum on the other (LV + S) decreased from 0.46 ± 0.016 g to 0.29 ± 0.006 g in males and from 0.63 ± 0.03 g to 0.24 ± 0.008 g in females, *n* = 6, *p* < 0.001. Additionally, PAH increased basal contractility in female groups, and each of the diet allocations (l-ARG, ND, and mixed) were found to restore contractility to control values. All diet protocols in male and female restored decreased responsiveness of the myocardium to norepinephrine in hearts obtained from rats with PAH and prevented vascular changes observed in pulmonary hypertension (thickness of blood vessels and cell infiltration).

**Conclusion:**

Our study suggests that l-arginine, nicotinamide or both play a positive role in right ventricle function or the process reducing pulmonary vascular remodeling especially in a gender-independent way.

## Introduction

Pulmonary arterial hypertension (PAH) is a clinical condition caused by a multitude of factors, and affects the functioning of the myocardium and pulmonary circulation [1]. Progression of this disease leads to the narrowing of pulmonary arteries, elevation of pulmonary resistance (PVR), right ventricle remodeling (cardiomyocyte necrosis) and reduction of right ventricular ejection fraction [2]. Finally, it results in acute or chronic heart failure and the death of the patient [3]. According to the literature, PAH is defined as an elevated mean pulmonary artery pressure > 25 mmHg at rest and > 30 mmHg during exercise and increased pulmonary vascular resistance [4]. Interestingly, the pressure in the right atrium and cardiac output (CO) are one of the most important indicators predicting the survival rate of PAH patients. Currently, therapeutic recommendations for PAH are focused on the pulmonary vessels and do not consider simultaneous treatment of myocardium dysfunction [5]. In this context, the use of sex hormones, especially progesterone, nicotinamide and l-arginine is promising [6–8]. Nicotinamide is known to have many biological and therapeutic properties, including antioxidant, anti-inflammatory, and proapoptotic [7]. Studies suggest nicotinamide has a protective effect on acute lung damage caused by ischemia, endotoxin or oxidative stress [8]. Recently, studies have shown that nicotinamide can inhibit poly (ADP-ribose) polymerase (PARP), which in response to free radicals increases rapidly, causing a decrease in NAD + and ATP (metabolic homeostasis). The role of nicotinamide in PARP inhibition is important in vascular smooth muscle cells (inhibits spasm), in myocardial cells or cancer cells as it maintains an appropriate level of ATP [9]. In addition, nicotinamide has been shown to regulate myocardial SUR2A receptors by increasing cardiac resistance to an ischemia/reperfusion reaction [10].

l-Arginine is a nitric oxide (NO) donor. Some diseases, for example atherosclerosis, hypercholesterolemia or pulmonary hypertension significantly reduce the level of NO [11, 12]. Vascular endothelial cells use l-arginine as a NO precursor for physiological processes such as: inhibition of myocyte proliferation, vascular remodeling, reduction of vascular wall tension, inhibition of leukocyte aggregation and platelet aggregation. In addition, NO activates the Ca^2+^/calmodulin complex, which has a cardioprotective and smooth muscle relaxant effect [13]. It can reduce the level of free radicals and minimize inflammation in the blood vessels. In the heart, it reduces pulmonary resistance [14], maintains a low right heart ventricle index, increases myocardial contractility, reduces reactions to oxidative stress, and increases myocyte relaxation [15]. Therefore, this study was designed to investigate whether the addition of ND and l-ARG to the standard diet, separately and in combination, would have an impact on changes to heart contractility and pulmonary vessel structure induced by pulmonary hypertension. The study design was based on a well-established model of pulmonary hypertension in male and female rats.

## Materials and methods

### Animals and experimental design

The protocol of our experimental study was approved by the Local Ethics Committee for Animal Experiments No 3, Medical University of Gdansk. All experimental procedures were performed on male and female Wistar albino rats with an initial body weight of 198 ± 3 g. The animals were maintained under a controlled temperature of 23 ± 2 °C, a relative humidity of 53 ± 2% and a fixed lighting cycle of 12 h light/night for 7 days before the start of experiment. The animals were divided into two per cage and separated by gender. The animals received AIN93G food (ssniff^®^Spezialdiäten GmbH, Soest, Germany) and water ad libitum throughout the entire study. Male (M) and female (F) rats were divided into 16 groups of 6 animals each, as follows:

- Control groups that received a standard diet: group-1 (male control-MC); group-2 (female control-FC); observational period 1–23 days;
- Groups that received l-arginine in their drinking water (2,5% solution/po from the 7th day): group-3 (male + l-ARG = MARG) and group-4 (female + l-ARG = FARG);
- Groups that received a nicotinamide-rich, diet (500 mg/kg of food/po from the 7th day): group-5 (male + ND = MND) and group-6 (female +ND = FND);
- Groups that received both l-ARG and ND at the same time: group-7 (MARG + ND) and group-8 (FARG + ND);
- Groups with pulmonary arterial hypertension (PAH) induced by monocrotaline 60 mg/kg B.W./sc, applied on the 1st experiment day): group-9 (MPAH) and group-10 (FPAH);
- Groups with PAH that received l-ARG from the 7th day: group-11 (MPAH + ARG) and group-12 (FPAH + ARG);
- Groups with PAH that received ND from the 7th day: group-13 (MPAH + ND) and group-14 (FPAH + ND);
- Groups with PAH that received both l-ARG and ND at the same time: group-15 (MPAH + ARG + ND) and group-16 (FPAH + ARG + ND).

**Fig. 1:**
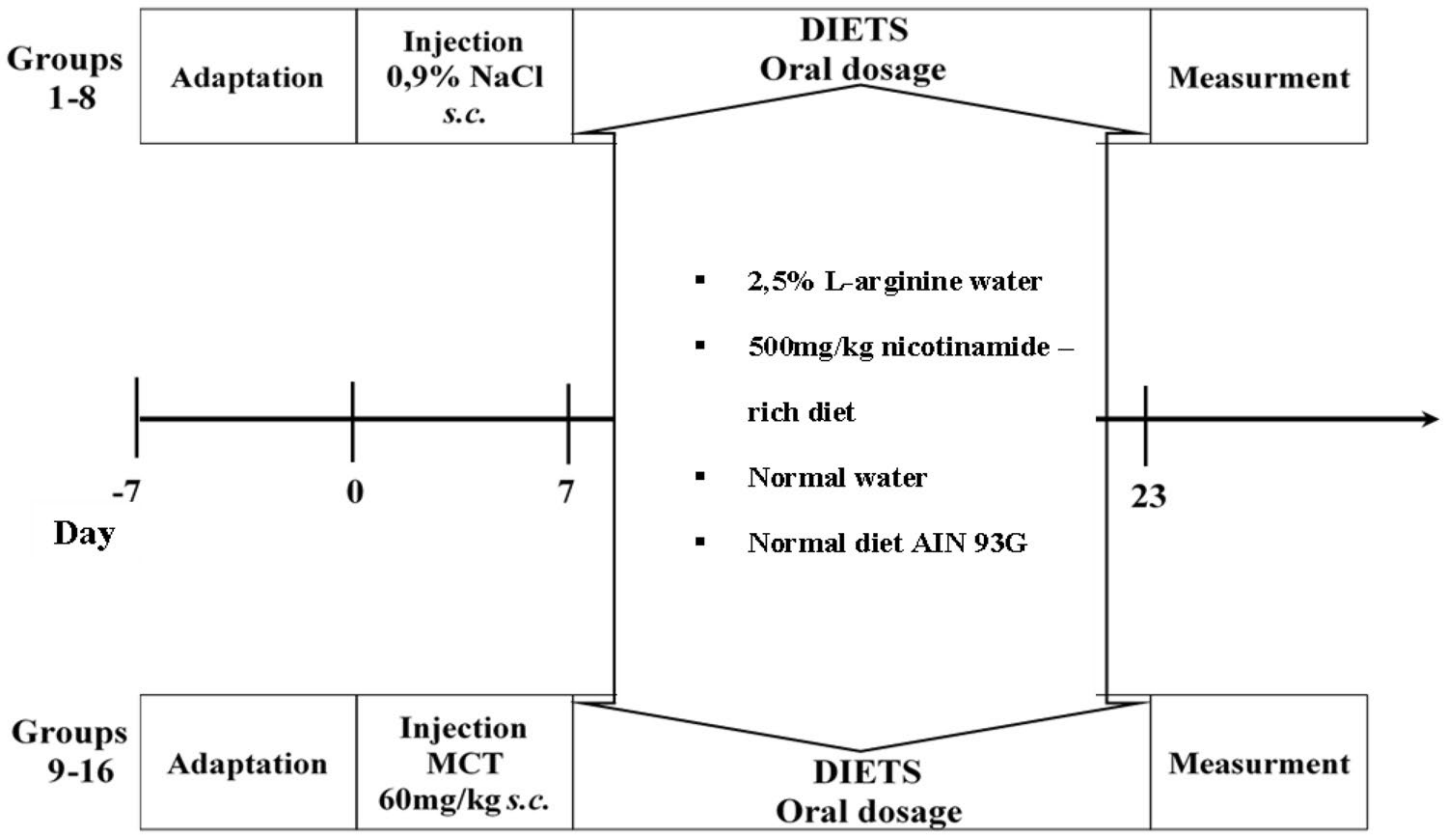
Range of experimental protocols used in this study

All control groups (1–8) were given an equal volume of 0.9% sodium chloride solution (1 ml/kg B.W./sc) on the 1st day of the 23-day observational period, instead of monocrotaoline (MCT) which was applied to experimental groups (9–16). MCT was dissolved in 1N HCl, and adjusted to a pH 7.4 with 1N NaOH. The diet regimen rich in l-arginine was four times the recommended dose for a rat [16]. The diet regimen rich in nicotinamide was 17 times higher than the recommended dose [17], however it did not exceed the maximal dose permitted in rats. The animals were weighed once a week. The entire experimental procedure is shown in Fig. 1.

After 23 days, the animals were sacrificed via overdose with sodium pentobarbital (60 mg/kg ip), blood samples were collected, the chest opened and the heart and lungs removed. The blood samples (1 ml from femoral arteries) were then centrifuged (3000 rpm) with citrate (v/v 9:1) for 15 min. The obtained plasma was stored at − 80 °C for further biochemical analysis. The heart was quickly removed and placed in a preparation dish with Krebs–Henseleit modified solution (KHs) and aerated with carbogen. To remove the thrombocytes and safely stop the contraction of the heart, the solution was cooled to 12 °C. Then, as quickly as possible, a papillary muscle > 3 mm long and < 1 mm in diameter from the right ventricle was isolated. The papillary muscle was placed in an organ bath. The lungs were placed in 8% of buffered formalin for later histopathological and morphometric analysis.

### Measurement of plasma level of nicotinamide

To detect the level of ND in plasma we used high-performance liquid chromatography with mass detection (LC/MS) in accordance with the previously developed method [18]. Blood samples for this analysis were collected on the 23rd day of the observational period in groups: 1, 2; 5, 6; 9, 10; 13, 14. The level of l-arginine in plasma was not able to be determined, as the compound is metabolized rapidly and the dose we used for supplementation had been proven to be effective [19].

### Measurement of organ mass

To rate the degree of right ventricle size changes in different experimental groups, the hearts were divided into two sections: the right ventricle (RV), and the left ventricle with the septum (LV + S), and each section was weighed separately (Fulton index) [20–23]. The normal ratio RV against LV + S was 0, 2. A result above 0, 3 was considered as being classified PAH [24]. Additionally, the weight of individual heart chambers to the final body weight (FBW) were evaluated for: the right ventricle (RV/FBW), the left ventricle with the septum (LV + S/FBW), and for the total weight of the heart to final body weight (HW/FBW).

### Measurement of force of contraction

Papillary muscle was excised from the right ventricle and placed into an organ bath of 2 ml (Steiert organ bath, type 813, DC temperature regulator, type 319, Hugo Sachs Elektronik, Germany). The tissue was perfused with KH solution with the following composition: NaCl 120.4; KCl 4.9; MgCl_2_ × 6H_2_O 0.6; CaCl_2_ 2.5; NaH_2_PO_4_ × H_2_O 1.0; NaHCO_3_ 15.3; glucose 11.5; sodium pyruvate 2.0. Constant rate of perfusion was approximately 8 ml/min and was maintained by means of a peristaltic pump (peristaltic pump, type 371, Unipan, Poland). Constant solution temperature was 37 °C ± 0.5. The solution was aerated with a gas mixture of 95% O_2_ and 5% CO_2_, the pH was kept constant at 74. The tissue was incubated for 60 min at 4 mN ± 0.12 of resting force (1 mN = 0.1 g), in contact with two silver electrodes for stimulation at 1 Hz, square wave current, 3 ms pulse duration, 2000 ms base cycle length, 20% amplitude above the threshold voltage, generated by the pacemaker with the insulating unit (ST-02, Experimetria, Hungary). *F*_c_ was measured with an isometric transducer (F-30, HSE, Germany) and a differential with measuring bridge type 336 (HSE, Germany). The results were recorded using a two-channel line recorder (Model 202, Cole-Parmer, United States). After 60 min stabilization, the papillary muscle was treated with noradrenaline in increasing, sequential concentrations from 0.1 to 100 μM.

### Histopathology and morphometric measurement of pulmonary arteries

The lungs obtained from the rats were preserved in 8% buffered formalin for histopathological and morphometric analysis. The tissues were stained with hematoxylin and eosin (H&E) to identify any structural changes. The preparations were examined under a light microscope (Carl Zeiss, type JenaMed, Germany) at 400 × magnification. Quantitative analysis included 15 fields of cross-sectional view of each case. Structural changes were interpreted and classified using a scale ranging from + (presence of damage) to +++ (very serious and extensive damage). A score of 0/+ indicated that in a given case, there was no (“0”) to some damage (“+”) observed. A single “0” sign indicated the normal appearance of tissue. Morphometric analysis was carried out at 400 × magnification using a set containing a light microscope, a digital camera (Olympus, type DP21, Japan) and a computer with software (cell^®^Sens Standard 1.7, Olympus, Japan). Two types of pulmonary arteries were selected for analysis: distal arterioles in the vicinity of the alveoli (vascular diameter from 30 to 100 μm) and arteries at the bronchioles (vascular diameter from 100 to 200 μm). We measured wall thickness of the selected artery from the inner lumen of the vessel to the outer wall of the vessel, in the thickest and thinnest part of the vessel (20 measurements in each case).

### Chemicals and diet

l-arginine, monocrotaline and noradrenaline were purchased from Sigma-Aldrich, St. Louis, MO, USA. Nicotinamide (nicotinamide-rich diet) was sourced from ssniff^®^ Sapsialdiäten GmbH, Soest, Germany.

### Statistical analysis

The force of contraction (*F*_c_) was expressed in mN (1 g = 10 mN). Morphometric measurement was expressed in μm. Each experiment result was evaluated as mean ± standard error (SEM) and statistical analysis was performed using STATISTICA version 13.3 (StatSoft^®^, Inc. 2017, https://www.statsoft.com). The multi-way ANOVA was applied to compare and evaluate data from more than two independent groups along with the multiple comparison Neuman–Keuls test, where deemed necessary. A difference of *p* < 0.05 was considered statistically significant.

## Results

### The serum levels of nicotinamide metabolites

We determined the plasma concentrations of four ND metabolites. It was observed that gender significantly differentiates the metabolite value of Met-2PY (*F*_1,12_ = 15.40, *p* = 0.002) and the type of diet administered significantly affects the value of Met-2PY (*F*_3,12_ = 28.88, *p* = 0.000009), and a significant interaction between gender × diet (*F*_3,12_ = 6, *p* = 0.006). Post hoc comparisons showed that the level of Met-2PY in male serum was higher (*p* = 0.0003) compared to the female group. Also, Met-4PY levels was significantly differentiates through gender (*F*_1,12_ = 13.82, *p* = 0.002) and the type of diet was administered significantly affects the value of Met4PY (*F*_3,12_ = 11.2, *p* = 0.0008), but no interaction was showed between gender × diet. Post hoc comparisons showed that the level of Met-4PY in male was higher (*p* = 0.0006) compared to the female group and only diets significantly differentiated Met-NA levels (*F*_3,12_ = 19.76, *p* = 0.00006). Others data were shown in Table 1.

**Table 1:** Effects of nicotinamide-rich diet on serum level of metabolites nicotinamide in pulmonary arterial hypertension male and female rats induced by monocrotaline

Groups	Met-NA		Met-2PY		Met-4PY		4-PYR	
	(♂) Male	(♀) Female	(♂) Male	(♀) Female	(♂) Male	(♀) Female	(♂) Male	(♀) Female
μmol/L								
Control	29.13 ± 10.59^c,j^	23.6 ± 4.49^a,g^	0.26 ± 0.12^e,i^	0.58 ± 0.11^b^	3.14 ± 0.61^b,f^	1.62 ± 0.041	1.68 ± 0.87	1.04 ± 0.33
ND	570.85 ± 78.63	476.07 ± 66.13	13.29 ± 1.61	7.21 ± 1.7	7.68 ± 0.85	4.53 ± 1.22	1.1 ± 0.32	1.02 ± 0.18
PAH	12.945 ± 0.22^a,k^	17.64 ± 2.78^b^	0.31 ± 0.14^d,h^	0.19 ± 0.01	2.42 ± 0.027^a,f^	1.665 ± 0.35	0.95 ± 0.09	1.23 ± 0.28
PAH + ND	599.59 ± 168.03	363.42 ± 41.66	15.72 ± 2.69	4.8 ± 0.37	7.55 ± 1.24	3.64 ± 0.91	1.92 ± 0.67	1.14 ± 0.2

*Met-NA N*-methylonicotinamide, *Met-2PY N*-methyl-2-pyridone-5-carboxamide, *Met-4PY N*-methyl-4-pyridone-3-carboxamide, *4-PYR* 4-pyridone-3-carboxamide-1-β-d-ribonucleoside. Each value represents the mean ± SEM, *n* = 2–3 rat per groups. Statistically significant differences was indicated by the symbols: ^a^*p* = 0.01, ^b^*p* = 0.02, ^c^*p* = 0.004, ^d^*p* = 0.0005, ^e^*p* = 0.0007 vs. ND male or female group; ^f^*p* = 0.01, ^g^*p* = 0.04, ^h^*p* = 0.004, ^I^*p* = 0.008, ^j^*p* = 0.0002, ^k^*p* = 0.0003 vs. PAH + ND male or female group

### The effects of l-arginine and nicotinamide on the final body weight (FBW) and heart weight (HW)

Table 2 showed that pulmonary hypertension reduced the FBW in 18% of all male groups, regardless of the type of diet provided. Interestingly, the FBW reduction induced by pulmonary hypertension in female rats was smaller than that observed in male groups, with an average decrease of approximately 7%. Multivariate analysis of variance has shown a significant effect of gender (*F*_1,80_ = 2354.1, *p* = 0.0001), administered diets (*F*_7,80_ = 51.2, *p* = 0.0001) and a significant interaction between gender × diets (*F*_7,80_ = 22.9, *p* = 0.0001) on FBW. Post hoc test showed that FBW in male (*p* = 0.0001) was higher than in female. The multi-way ANOVA revealed that gender (*F*_1,80_ = 46.19, *p* = 0.00001) and administrated diets (*F*_7,80_ = 7.84, *p* = 0.00001) significantly differentiates HW/FBW and additionally has shown a significant interaction between gender × diets (*F*_7,80_ = 2.17, *p* = 0.04). Post hoc test showed that HW/FBW in male (*p* = 0.0001) was higher than in female. The analysis showed that gender (*F*_1,80_ = 115.8, *p* = 0.00001) and type of diet (*F*_7,80_ = 24.2, *p* = 0.00001) significantly differentiate the RV value and the occurrence of interaction (*F*_7,80_ = 3.3, *p* = 0.003), which means that the influence of gender depends on the type of diet used. The post hoc test showed that RV in males was significantly higher (*p* = 0.0001) than in females. In addition, it was shown that in PAH both in males and females there is a significant increase in RV value compared to the groups receiving diets in PAH. The multi-way ANOVA revealed that gender does not different the increase in RV/LV + S, but the values for females (*F*_1,80_ = 1.23, *p = *0.26) were higher than for males. The use of different diets significantly affects RV/LV + S (*F*_7,80_ = 48.76, *p = *0.00001). In addition, interactions between gender and type of diet were observed (*F*_7.80_ = 5.67, *p = *0.00002). The post hoc test showed the significant increase in RV/LV + S in PAH groups in males and females, and decrease of this parameter after the diet in PAH. The multi-way ANOVA revealed a significant impact of gender (*F*_1,80_ = 8.14, *p = *0.005) and type of treatment (*F*_7,80_ = 31.53, *p = *0.00001) and their interaction (*F*_7,80_ = 2.4, *p = *0.02) on RV/FBW. The post hoc test showed that the RV/FBW value is higher in males (*p = *0.005) than in females. The multi-way ANOVA revealed a significant effect of gender (*F*_1,80_ = 505.71, *p = *0.00001) and effect of diet type (*F*_7,80_ = 11.46, *p = *0.00001) on LV + S. However, there is no interaction between them. The post hoc test showed that the LV + S value is definitely higher in males (*p = *0.0001) than in females. It was also shown that gender (*F*_1,80_ = 57.16, *p = *0.00001) and the type of diet (*F*_7,80_ = 7.76, *p = *0.00001) used significantly differentiated the LV + S/FBW value and showed interactions between them (*F*_7,80_ = 7,80, *p = *0.01). Post hoc test showed a higher value in males (*p = *0.0001) than in females for this parameter(Table 2).

**Table 2:** Effect of nicotinamide and l-arginine, given alone and in combination, on changes in body, heart weights in pulmonary arterial hypertension male and female rats induced by monocrotaline

Groups	Control	ARG	ND	ARG + ND	PAH	PAH + ARG	PAH + ND	PAH + ARG + ND
Male								
Initial BW (g)	201.17 ± 1.19	198.5 ± 1.05	200.5 ± 1.33	200 ± 1.59	200.16 ± 1.44	201.16 ± 1.74	198.5 ± 2.66	198.5 ± 1.23
Final BW (g)	332.67 ± 2.7	340 ± 2.8	338.17 ± 2.1	335.5 ± 3.2	276.16 ± 2.9^c,f,i^	321 ± 3.5^a,l,o^	302.67 ± 2.4^c,f,i,o^	306.5 ± 4.2^c,f,i,l,o^
HW/FBW (g/kg)	2.74 ± 0.02	2.75 ± 0.15	2.91 ± 0.08	2.74 ± 0.03	3.28 ± 0.05^b^	3.05 ± 0.08^a,d^	3.16 ± 0.08	3.08 ± 0.11
RV (g)	0.17 ± 0.004^s^	0.21 ± 0.017^q^	0.19 ± 0.001^q^	0.19 ± 0.004^r^	0.28 ± 0.008^c,f,i,l^	0.24 ± 0.014^n^	0.20 ± 0.006^o^	0.21 ± 0.009^o^
RV/LV + S (g/g)	0.24 ± 0.008	0.29 ± 0.026	0.25 ± 0.006	0.26 ± 0.040	0.46 ± 0.016^c,f,i,l^	0.33 ± 0.026^o^	0.25 ± 0.014^o^	0.29 ± 0.006^o^
RV/FBW (g/kg)	0.52 ± 0.014^q^	0.61 ± 0.054	0.58 ± 0.071	0.57 ± 0.085	1.04 ± 0.037^c,f,i,l^	0.75 ± 0.046^o^	0.66 ± 0.017^o^	0.70 ± 0.037^o^
LV + S (g)	0.74 ± 0.005	0.73 ± 0.04	0.78 ± 0.025	0.73 ± 0.012	0.62 ± 0.004^a^	0.73 ± 0.026^n^	0.75 ± 0.026^n^	0.73 ± 0.018^m^
LV + S/FBW (g/kg)	2.21 ± 0.015	2.14 ± 0.119	2.32 ± 0.075	2.17 ± 0.027	2.24 ± 0.025	2.29 ± 0.08	2.49 ± 0.08^a,g,m^	2.38 ± 0.08^a,j,m^
Female								
Initial BW (g)	197.83 ± 1.1	196.83 ± 0.9	196 ± 1.06	195.83 ± 1.16	197.67 ± 0.98	198.67 ± 1.02	198.17 ± 1.68	196.67 ± 1.58
Final BW (g)	270.17 ± 1.4	253.5 ± 2.07^c^	245.83 ± 3.23^c^	254.33 ± 3.13^c^	242.83 ± 2^c^	251 ± 3.12^c^	247.33 ± 1.23^c^	242.83 ± 3.06^c,j^
HW/FBW (g/kg)	2.48 ± 0.03	2.56 ± 0.07	2.73 ± 0.06	2.52 ± 0.06	2.78 ± 0.11	2.5 ± 0.13	3.08 ± 0.19^b^	2.49 ± 0.07
RV (g)	0.12 ± 0.003^t^	0.12 ± 0.001^t^	0.14 ± 0.001	0.13 ± 0.006	0.26 ± 0.017^c,f,i,l^	0.15 ± 0.024^o^	0.18 ± 0.02^o^	0.12 ± 0.005^o^
RV/LV + S (g/g)	0.23 ± 0.007	0.24 ± 0.010	0.27 ± 0.008	0.26 ± 0.015	0.63 ± 0.0035^c,f,i,l^	0.31 ± 0.051^o^	0.30 ± 0.018^o^	0.24 ± 0.009^o^
RV/FBW(g/kg)	0.46 ± 0.010^t^	0.50 ± 0.007^t^	0.58 ± 0.009	0.52 ± 0.026^t^	1.07 ± 0.075^c,f,i,l^	0.59 ± 0.01^o^	0.73 ± 0.08^o^	0.48 ± 0.026^o,t^
LV + S (g)	0.54 ± 0.004	0.52 ± 0.015	0.53 ± 0.014	0.51 ± 0.018	0.41 ± 0.014^c,f,i,l^	0.47 ± 0.015^a,n^	0.58 ± 0.02^o^	0.49 ± 0.02^b,n^
LV + S/FBW (g/kg)	2.01 ± 0.023^n,t^	2.06 ± 0.071^n,t^	2.15 ± 0.055^o^	1.99 ± 0.057^n,t^	1.70 ± 0.054	1.89 ± 0.061^m,w^	2.29 ± 0.1^o^	2.01 ± 0.048^n,t^

*BW* body weight, *FBW* final body weight, *HW* heart weight, *RV* right ventricle, *LV + S* left ventricle and septum. Each value represents the mean ± SEM of six animals from each experimental groups. Statistically significant differences are indicated by the symbols: ^a^*p* < 0.05 or ^b^*p* < 0.01 or ^c^*p* < 0.001 vs. Control group; ^f^*p* < 0.001 vs. ARG; ^g^*p* < 0.05 or ^I^*p* < 0.001 vs. ND; ^l^*p* < 0.001 vs. ARG + ND; ^m^*p* < 0.05 or ^n^*p* < 0.01 or ^o^*p* < 0.001 vs. PAH; ^q^*p* < 0.05 or ^r^*p* < 0.01 or ^s^*p* < 0.001 vs. PAH + ARG; ^t^*p* < 0.05 vs. PAH + ND

### The effects of noradrenaline on the force of contraction (*F*_c_)

**Fig. 2:**
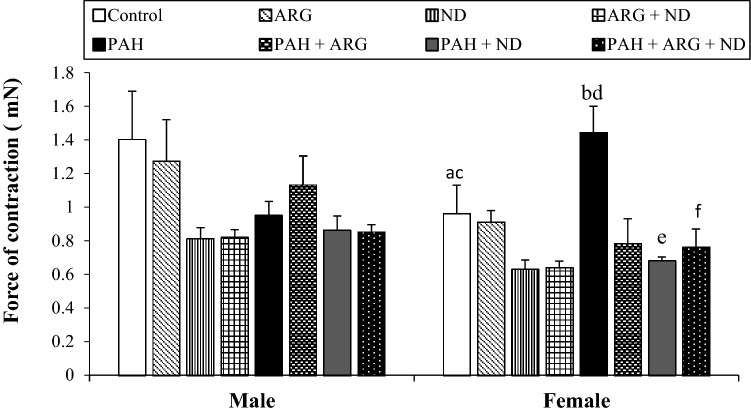
The effect of nicotinamide-rich diet, l-arginine diet and pulmonary arterial hypertension (PAH) on the resting force of contraction (*F*_c_) of isolated right papillary muscles from male and female rats. Each value represents the mean ± SEM of six animals from each experimental groups. Statistically significant differences are indicated by the symbols: ^a^*p = *0.01, ^b^*p = *0.007 vs. ND; ^c^*p = *0.01, ^d^*p = *0.008 vs. ARG + ND; ^e^*p = *0.01, ^f^*p = *0.039 vs. PAH

**Fig. 3:**
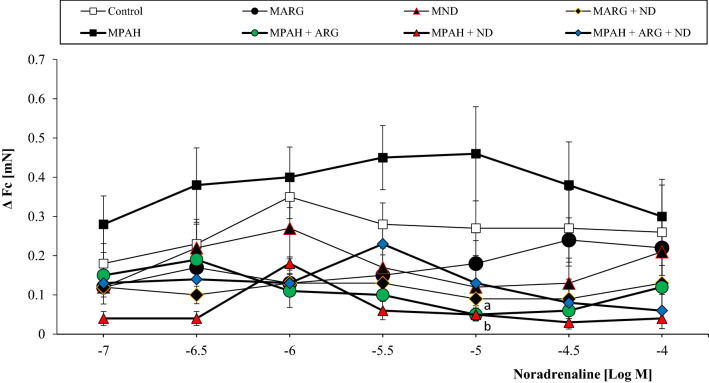
The effects of noradrenaline on the force of contraction of isolated papillary muscles from male rats fed with a nicotinamide-rich diet and l-arginine in control groups and groups with PAH. Each value represents the mean ± SEM of six animals from each experimental groups. Statistically significant differences are indicated by the symbols: ^a^*p = *0.036, ^b^*p = *0.039 vs. MPAH

**Fig. 4:**
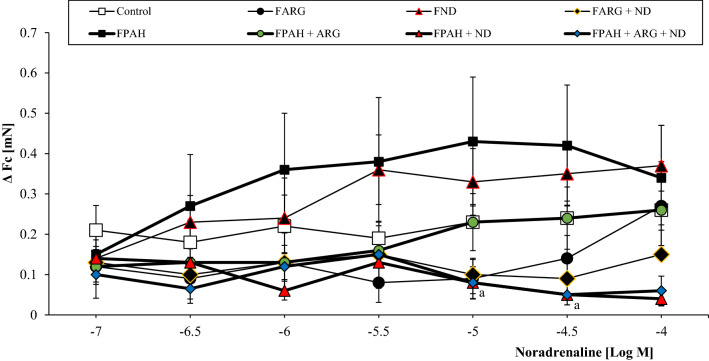
The effects of noradrenaline on the force of contraction of isolated papillary muscles from female rats fed with a nicotinamide-rich diet and l-arginine in control groups and groups with PAH. Each value represents the mean ± SEM of six animals from each experimental groups. Statistically significant differences are indicated by the symbols: ^a^*p = *0.03 vs. FPAH

First, we compared the resting force (or basal contractility induced by electrical stimuli, see “Materials and methods”) of isolated papillary muscle from different experimental groups (Fig. 2). The multi-way ANOVA revealed a significant difference in the impact of gender (*F*_1,80_ = 5.62, *p = *0.02) and the type of diet (*F*_7,80_ = 4.3, *p = *0.0004) used and their interaction (*F*_7,80_ = 2.14, *p = *0.04) on resting force. We found that the addition of diets and pulmonary arterial hypertension had an effect on resting force causing a significant reduction in basal contractility compared to PAH. Post hoc comparisons showed that males have higher *F*_c_ (*p = *0.02) than females. There were significant differences of the force of contraction in females from the Control group (*p = *0.01 and *p = *0.01) and PAH (*p = *0.007 and *p = *0.008) compared to the ND group and ARG + ND, respectively. Administration of ND (*p = *0.01) and ARG + ND (*p = *0.039) in pulmonary hypertension significantly reduced *F*_c_ compared to PAH only in female rats. The next step was to examine the effect of pharmacological stimulation on contraction strength. We used noradrenaline as a potent stimulator of α and β1 adrenoceptors, strongly expressed in the hearts of rats. The multi-way ANOVA revealed that the type of diet administered (*F*_7,560_ = 27.14, *p = *0.00001) significantly differentiated the effect of noradrenaline on the force of contraction of the heart muscle and significant gender-diet interactions (*F*_7,560_ = 2.88, *p = *0.005) were demonstrated. The addition of ND and l-ARG, separately and in combination, decreased the reaction of papillary muscle to noradrenaline either in control groups or in groups with PAH. However, the reduction of noradrenaline effects by ND and l-ARG was much stronger in groups with PAH (Figs. 3, 4).

### The effects of nicotinamide and l-arginine on pulmonary vascular remodeling induced by PAH in male and female rats

**Fig. 5:**
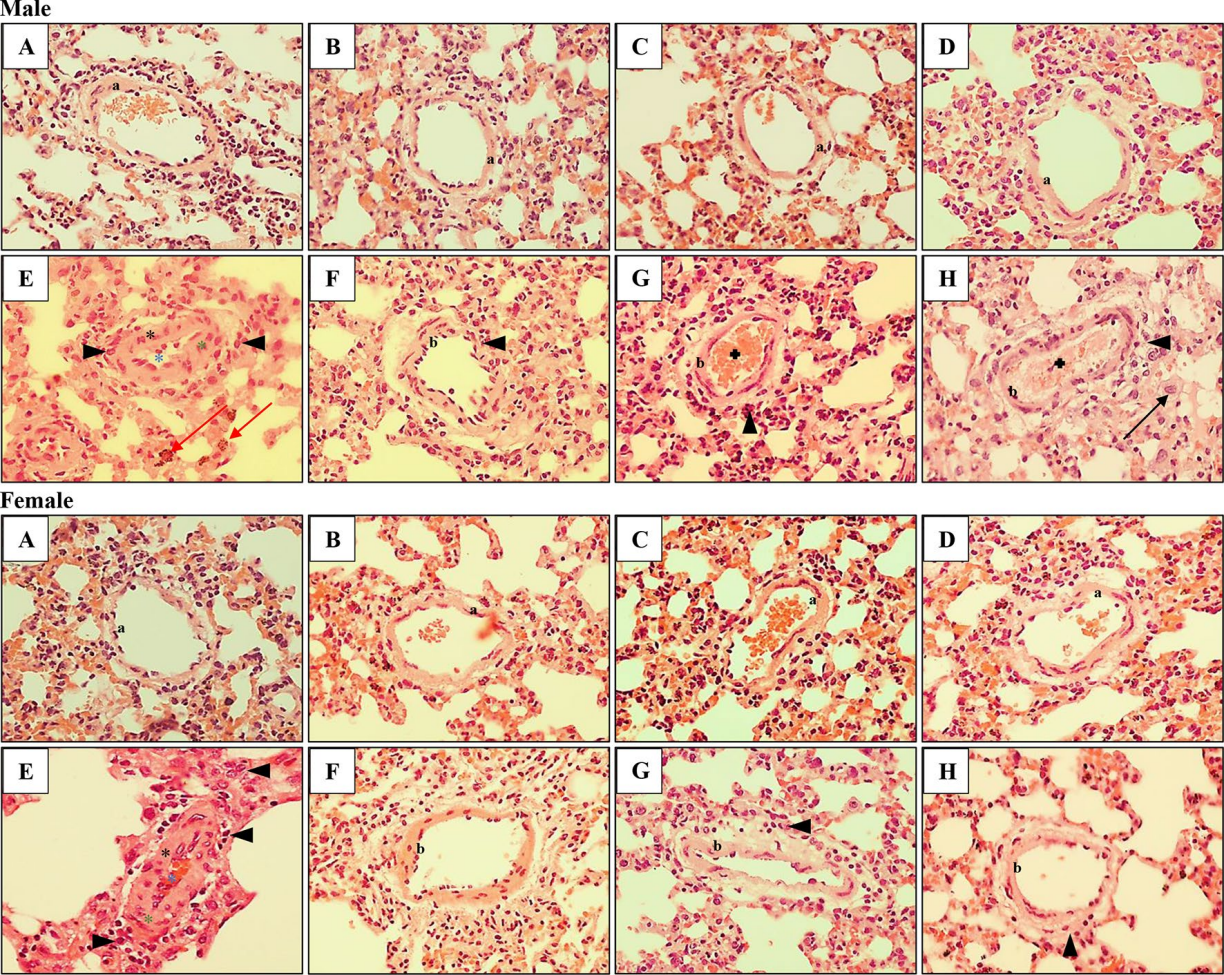
Histological analyses of male and female rat lung tissue. Effect of ARG and ND, administered separately and in combination, on PAH-induced changes in the thickness of the pulmonary arteries located in the area of the alveoli in male (♂) and female (♀) rats. Normal (a) pulmonary artery structures in Control, ARG, ND and ARG + ND male and female groups (**a**–**d**, respectively). Severely affected pulmonary arteries with PAH (black asterisk), thickening of the vessels walls (green asterisk) and narrowing of the vessels light (blue asterisk), inflammatory cell infiltration (arrowhead) in male and female (**e**) groups and congestion (red arrow) in male PAH (**e**) group. Normed pulmonary artery structures (b) in PAH + ARG male and female groups (**f**). Additional inflammatory cell infiltration (arrowhead) in PAH + ARG male group (**f**). Normed pulmonary artery structures (b) and inflammatory cell infiltration (arrowhead) in PAH + ND female (**g**) group and mild vasocongestion (plus) in PAH + ND male group (**g**). Normed (b) pulmonary arteries and inflammatory cell infiltration (arrowhead) in PAH + ARG + ND female group (**h**), and also vasocongestion (plus) and macrophages (black arrow) in PAH + ARG + ND male group (**h**); H&Ex400

**Fig. 6:**
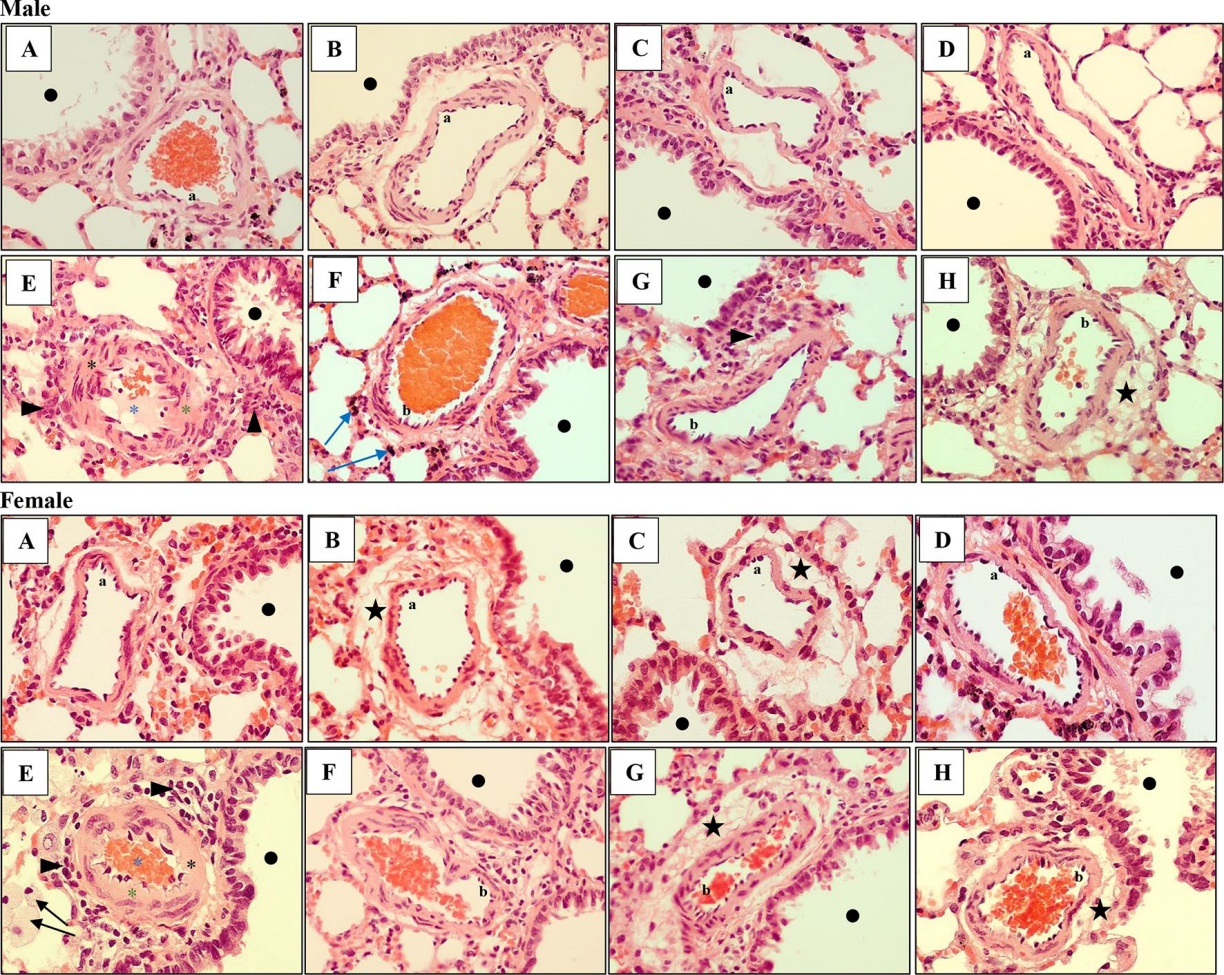
Histological analyses of male and female rat lung tissue. Effect of ARG and ND, administered separately and in combination, on PAH-induced changes in the thickness of the pulmonary arteries located in the bronchial area (black dot) in male (♂) and female (♀) rats. Normal (a) pulmonary artery structures in Control, ARG, ND and ARG + ND male and female groups (**a**–**d** respectively). However, mild perivascular edema (black star) in ARG and ND female groups (**b**, **c**). Severe changed pulmonary arteries with PAH (black asterisk), thickening of the vessel walls (green asterisk) and narrowing of the vessels light (blue asterisk), inflammatory cell infiltration (arrowhead) in male and female (**e**) groups and macrophages (black arrow) in female PAH group (**e**). Normed pulmonary artery structures (b) in PAH + ARG male and female groups (**f**). Additional perivascular hemorrhages (blue arrow) in PAH + ARG male group (**f**). Normed pulmonary artery structures (b) and inflammatory cell infiltration (arrowhead) in PAH + ND male group (**g**) and perivascular edema (black star) in PAH + ND female group (**g**). Normed (b) pulmonary arteries and perivascular edema (black star) in PAH + ARG + ND male and female groups (**h**); H&Ex400

**Fig. 7:**
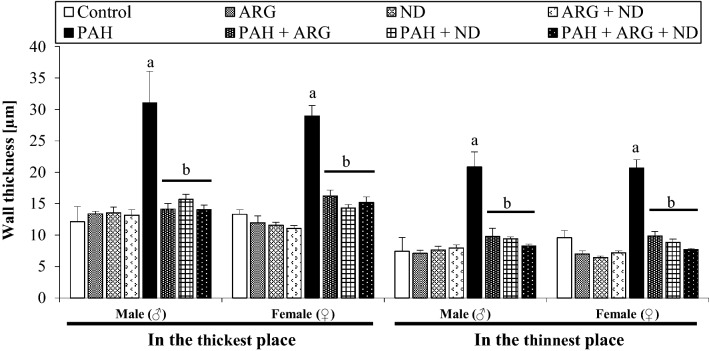
Effect of nicotinamide and l-arginine given alone and in combination in male (♂) and female (♀) rats with PAH. Thickness of pulmonary vascular wall located in the area of the alveoli was measured. Each value represents the mean ± SEM, *n* = 3-6 rats/group. Statistically significant differences are indicated by the symbols: ^a^*p = *0.0001 vs. Control, ARG, ND or ARG + ND group; ^b^*p = *0.0001 vs. PAH

**Fig. 8:**
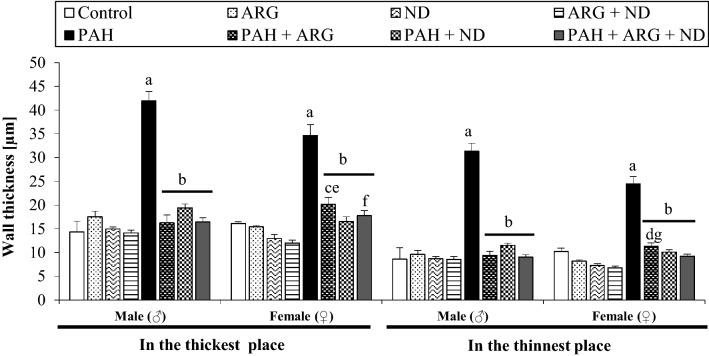
Effect of nicotinamide and l-arginine given alone and in combination in male (♂) and female (♀) rats in experimental pulmonary arterial hypertension (PAH). Thickness of pulmonary vascular wall located in the bronchial area. Each value represents the mean ± SEM, *n* = 3–6 rats/group. Statistically significant differences are indicated by the symbols: ^a^*p = *0.0001 vs. control, ARG, ND and ARG + ND; ^b^*p = *0.0001 vs. PAH; ^c^*p = *0.004, ^d^*p = *0.032 vs. ND; ^e^*p = *0.0005, ^f^*p = *0.038, ^g^*p = *0.009 vs. ARG + ND

Table 3 presents data related to the morphologic changes in pulmonary vessels induced by PAH in male and female rats in both the absence and presence of l-arginine and nicotinamide. As Figs. 5 and 6 show, PAH induced necrotic lesions, perivascular edema, interstitial pneumonia, atelectasis, platelets plug, and endovascular infiltrations with macrophages, which were observed in both, males and females. It is important to note that the small changes identified in pulmonary arterioles (diameter up to 0.3 mm) are progressive occlusions, observed in more than 90% of cases. Additionally, in more than 80% of females with PAH, pulmonary edema was observed, however with a smaller overgrowth of the medial vascular layer when compared to males with PAH. The addition of l-ARG reduced the aforementioned changes (in males in over 70% of cases, and in females in over 60% of cases). On the other hand, aside from the reduction of some morphological changes induced by pulmonary hypertension, the addition of ND induced intravascular inflammation (10% of males, 60% of females), as well as infiltration with macrophages and angioedema (10% of males and 40% of females). Finally, a combined l-ARG + ND diet decreased the level of changes appearing in PAH in more than 70% of the studied cases and also reduced the abnormalities associated with the administration of nicotinamide alone, in both males and females. The multi-way ANOVA showed that the type of diet administered significantly differentiates the pulmonary arterioles among the follicles in the thickest (*F*_7,59_ = 39.06, *p = *0.00001) and in the thinnest (*F*_7,59_ = 39.06, *p = *0.00001) place. Gender does not significantly affect arterial thickness. There was also no interaction of gender x diet type. In contrast, multi-way ANOVA showed that the type of diet administered significantly differentiates the pulmonary arterioles among the bronchioles in the thickest place (*F*_7,58_ = 79.59, *p = *0.000001) and significant interaction between gender and diet type (*F*_7,58_ = 4.007, *p = *0.001) was demonstrated. The multi-way ANOVA revealed that gender (*F*_1,58_ = 8.84, *p = *0.004) and diet type (*F*_7,58_ = 117.81, *p = *0.00001) significantly differentiate arterioles among the bronchioles in the thinnest place. A significant interaction of gender x diet type (*F*_7,58_ = 4.91, *p = *0.0002) has also been shown. Post hoc test comparisons showed that females had a smaller increase in arterial wall thickness at the thinnest place than males. It should be noted that PAH significantly increased the thickness of small pulmonary arteries located around the alveoli and bronchi, and the addition of l-ARG and ND, separately and jointly, reduced this wall thickness in approximately 50% of cases (Figs. 7, 8).

**Table 3:** The effect of nicotinamide and l-arginine diets on the morphological change of pulmonary arteries of male and female rats in induced pulmonary arterial hypertension

Lung damage	Changes in pulmonary arteries		Vasculitis the wall of vessel		Hemorrhages and congestion	Edema	Macrophages
Groups	Thickening of the vessel walls	Narrowing of the vessel’s light	Around	In			
Male							
Control	0	0	0	0	0	0	0
ARG	0	0	0	0	0	0	0
ND	0	0	0	+	0	+	0
ARG + ND	0	0	0	0	0	+	0
PAH	+++	++/+++	++	+++	++	++	++/+++
PAH + ARG	0	0	0	+	0	+	+
PAH + ND	+/++	0	+	+/++	0	++	++
PAH + ARG + ND	+	0	0/+	0/+	0	++	+
Female							
Control	0	0	0	0	0	0	0
ARG	0	0	0	0	0	+	0
ND	0	0	0	0/+	+	+	0
ARG + ND	0	0	0	0/+	0	0/+	0
PAH	++/+++	+/++	+++	+/++	++	+++	+++
PAH + ARG	+	0	+	+	0	+/++	+
PAH + ND	+	0	0	+/++	0	+++	0
PAH + ARG + ND	+	0	+	0	0	+	+

Quantitative analysis related to histopathological changes in lung tissue of male and female MCT-treatment rats and included 15 fields of view of the cross-section in a given case. Interpretation of results in the range of + (presence of a given damage) to +++ (very serious and extensive damage). The minimal change was expressed by 0/+ means that the character 0 (there was no damage), the sign + (there was damage)

## Discussion

Given that pulmonary arterial hypertension (PAH) is an extremely complex disease with a poor prognosis [25], further investigation in this clinical field is essential. This study is the first to investigate the impact of oral administration of l-arginine (l-ARG) and nicotinamide (ND), separately and in combination, on myocardial function and histopathological changes in pulmonary arteries during the development of PAH in a gender-dependent way. The major novel finding of this study is that the combination of the oral supplements l-ARG and ND prevented right ventricle enlargement and vascular changes in small pulmonary arteries induced by pulmonary hypertension, especially in females. Additionally, the contractility of the right ventricle and the reaction to a catecholamine (noradrenaline) was preserved and restored to the normal, control level.

We used a well-established model of PAH induced by monocrotaline (MCT) in rats [26]. MCT is a macrocyclic pyrrolizidine alkaloid obtained from seeds of a *Crotalaria spectabilis* plant. The exact mechanism of action of this compound is not known. After administration of MCT, a “monocrotaline syndrome” appears which is manifested by: acute necrotic inflammation of the pulmonary arteries and hypertrophy of the right ventricle [27]. In this study, MCT was administered at a dose of 60 mg/kg B.W., with an observational period of 23 days to allow full development of pathological changes in heart and pulmonary circulation. Higher doses may contribute to hepato-, cardio-, and nephrotoxicity and are associated with a higher mortality rate [27]. The use of this model was confirmed to be effective by Meyrick et al., who observed the remodeling of distal pulmonary arteries after 3 days and RV hypertrophy 7–14 days after MCT was administered [28]. Additionally, significant reductions of the FBW (especially in male rats) [29], increased levels of catecholamines and the enlargement of the right ventricle (especially in female rats) have been reported [30, 31]. Finally, MCT may induce oxidative stress, bringing high ROS production in the lungs and in RV and, as a consequence, producing cytotoxicity in the endothelial cells of pulmonary arteries [32].

As a result of 16-day supplementation with l-ARG and ND, separately or in combination, RV weight, RV/LV + S and RV/FBW ratios were found to have been restored, in both males and females, when compared to groups with PAH, with particular improvement noted in females. This effect was particularly visible when both compounds, l-ARG and ND were co-administered. This is consistent with previously published data showing that the use of l-arginine significantly reduced PAH symptoms [33]. On the other hand, the protective effect of nicotinamide in males has been confirmed in studies of acute lung injury, where this compound, in a dose-dependent manner, weakened the action of a strong neutrophil activator [8]. We observed that a ND-rich diet had a better effect on females than males when considering RV weight alone and in relation to the final body weight, in comparison to groups with PAH. Alternatively, l-ARG alone and l-ARG combined with ND prevented vascular changes induced by pulmonary hypertension in more male than female rats. The mechanism for the observed protective effects of l-ARG and ND may be related to the abolition of oxidative stress, reduction of inflammation and, as a consequence, a decrease in iNOS activity [34]. It is worth noting that in the FPAH + ND group, the histological picture was definitely more unfavorable than the corresponding control group and even the PAH group (observed more extensive inflammation and edema). This correlates with visible RV hypertrophy in this female group. A possible explanation could be a non-specific interaction between ND and MCT, or this substance could be hepatically metabolized in a different way in female as compared to male groups treated with ND [35].

Another unexplained study result related to the reduced (non-significant) resting (basal) force of contraction (*F*_c_) in males with pulmonary hypertension. The possible reason for this could be attributed to the effects of monocrotaline on the β_1_-adrenergic receptor signaling pathway, subsequently decreasing the production of cAMP content, as previously noted [36, 37]. Conversely, in the group of females with PAH, a significant increase of the resting force of contraction was observed. The cause of this gender discrepancy may be associated with the protective effects of estrogen in females, as well as the potential for males to experience a greater enlargement of the right ventricle during the development of PAH. This is consistent with previously published data on the strong cardioprotective effects of estrogen in different clinical situations [26, 38].

Our data clearly demonstrated that the addition of l-ARG and ND, separately or in combination, restored resting *F*_c_ to the control values in both male and female rats with PAH.

l-Arginine as a precursor of NO, exerts a negligible inotropic effect on the myocardium, on vasorelaxation as well as inhibits platelet activity under standard conditions [14, 15]. However, PAH induces endothelial dysfunction, which leads to the limited synthesis of NO.

Nicotinamide, is a cytoprotective compound with anti-inflammatory properties, which can also modulate the SUR2A subunit of ATP-dependent potassium channels in cardiomyocytes [10, 39]. It may play an important role in cardioprotection and may improve myocardial efficiency by inhibiting poly (ADP-ribose) and preventing the inhibition of mitochondrial respiration whilst simultaneously maintaining the balance of ATP in cells [8]. In addition, nicotinamide has anti-inflammatory properties resulting from the inhibition of iNOS synthase and the ability to remove free radicals [34].

In this study, we also examined the adrenergic responsiveness of right ventricular muscle under different experimental settings, using noradrenaline (NA), as the agonist of α and β_1_ adrenergic receptors. We observed the positive inotropic action of NA in all control and experimental groups, and observed a slight tendency of groups with PAH to have increased reaction to NA, which was restored to the control values by the addition of l-ARG, ND or l-ARG + ND. It is important to note that ND alone strongly increased responses to noradrenaline in the control female group, but decreased it in the group with pulmonary hypertension. Further research is needed to clarify the mechanism of the above-mentioned phenomenon.

To conclude, our study clearly showed that combined administration of l-arginine and nicotinamide has protective effects on vascular remodeling and myocardium contractility in PAH in a gender-independent way. This is of clinical interest considering a fact that female patients predominate this condition (4:1) [35]. However, the effects of these diets in humans with PAH are still unknown. To find out the clinical efficacy of this supplementation in PAH, further studies are meaningful and necessary, hopefuly, providing more options for the treatment of this serious disease.
